# Unique Presentation of Leukemic Cutaneous CD3/TCR- Phenotype T-Cell Lymphoma with Complete Remission after Allogeneic Stem Cell Transplantation

**DOI:** 10.4274/tjh.2016.0395

**Published:** 2017-06-01

**Authors:** Hatice Şanlı, Bengü Nisa Akay, Seçil Saral, Aylin Okçu Heper, Pervin Topçuoğlu

**Affiliations:** 1 Ankara University Faculty of Medicine, Department of Dermatology and Venereology, Ankara, Turkey; 2 Ankara University Faculty of Medicine, Department of Pathology, Ankara, Turkey; 3 Ankara University Faculty of Medicine, Department of Heamatology, Ankara, Turkey

**Keywords:** mycosis fungoides, Ichthyosiform, Sézary syndrome, Anaplastic, CD3/TCR-

## To The Editor,

**Figure 1:**
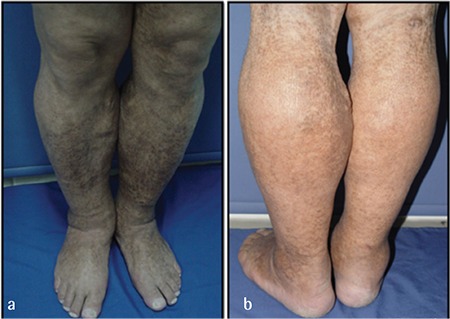
Ichthyotic plaques over anterior surface (a), and posterior surface (b) of lower extremities, edema and varicose dilatations of superficial veins are visible.

A 49-year-old male was admitted to our department with a 3-year history of pruritus and severe xerosis. Dermatological examination revealed squamation and ichthyosis with dark lamella involving the trunk and extremities (Figure 1). Physical examination revealed bilateral lower extremity edema.

Histopathological examination of the ichthyotic skin showed perivascular dermal lymphoid cell infiltration in the superficial dermis and nodular and dense lymphoid cellular infiltration in the deep dermis. Lymphoid cells were CD3^+^ and CD20- with a loss of CD7 expression. There were numerous (>25%) large, anaplastic cells with CD30 positivity among the lymphoid infiltrate. Clonal expansion of T cells in the lesional skin was demonstrated.

Complete blood count revealed 61.5x10^9^/L (reference range: 4.5-11) leukocytes with 15% Sezary cells. The axillary lymph node was consistent with N3 mycosis fungoides (MF) involvement. Thorax, abdominal, and pelvic computed tomography was normal. Bone marrow biopsy revealed involvement with positive clonality.

Flow cytometric analysis of the peripheral blood revealed the CD3/TCR complex in only 10% of the T cells. The CD4/CD8 ratio was 23 among T cells lacking CD3 expression and 1.6 among CD3^+^ cells.

The patient met the international criteria for Sezary syndrome [(SS); stage IVB, T4N3M1B2] and was diagnosed with ichthyosiform MF with large cell transformation with atypical flow cytometric phenotype [1,2]. According to the 2014 National Comprehensive Cancer Network Clinical Practice Guidelines [2], treatment was initiated as extracorporeal photopheresis, interferon-alpha-2a 3, and psoralen-UVA as first-line treatment without any response. The patient was unresponsive to polychemotherapy with gemcitabine and cisplatin and also three cycles of pralatrexate treatment. The patient underwent allogeneic hematopoietic peripheral stem cell transplantation (allo-HSCT) with an ablative conditioning regimen of cyclophosphamide (120 mg/kg) and total body irradiation (12 Gy) from an HLA-identical sibling donor. Cyclosporine A plus short-term methotrexate was given for graft-versus-host disease (GVHD) prophylaxis. Chronic sclerodermoid GVHD developed 1 year later and extracorporeal photopheresis was started for GVHD with complete response. After allo-HSCT, clonal T cells disappeared and skin lesions resolved completely.

There are only 5 patients showing negative TCR/CD3 complex in the literature and these patients were reported to have SS and atypical skin lesions, mainly non-erythrodermic leukemic variants, papuloerythroderma of Ofuji, prurigo nodularis, atopic dermatitis, papular xanthomatosis, and poikiloderma atrophicans vasculare-like lesions [3,4,5,6,7].

Allo-HSCT has been proven to be an effective therapy in MF/SS, demonstrating a decrease in the relapse rate and an overall increase in disease-free survival compared with conventional therapy. In a series of MF/SS transplants, Molina et al. [8] observed complete remission of skin lesions in 100% of patients after allo-HSCT. Duarte et al. [9] reported that 1 year after allo-HSCT, 42% of their patients remained in remission. Use of total skin electron beam as a debulking agent before conditioning with non-myeloablative allo-HSCT may reduce the severity of post-transplantation cutaneous GVHD [10].

In conclusion, our case is the first CD3-/TCR-SS patient presenting with generalized ichthyosis. All the patients with this immunophenotype are reported to have SS with intriguing skin lesions. These patients may require early initiation of more aggressive therapies. In our patient, allo-HSCT treatment resulted in cure and remission in a follow-up period of 3 years.
